# Transjugular intrahepatic portosystemic shunt-induced hemolysis in a non-cirrhotic patient: a case report

**DOI:** 10.1186/s13256-023-03953-7

**Published:** 2023-06-15

**Authors:** Michele Barnhill, Blanca Lizaola-Mayo, Sailendra G. Naidu, Surbhi Shah, David M. H. Chascsa

**Affiliations:** 1 Transplant Center, Mayo Clinic, 5777 E. Mayo Blvd, AZ 85054 Phoenix, USA; 2 Division of Gastroenterology and Hepatology, Mayo Clinic, Phoenix, AZ USA; 3 Division of Interventional Radiology, Mayo Clinic, Phoenix, AZ USA; 4 Division of Hematology and Medical Oncology, Mayo Clinic, Phoenix, AZ USA

**Keywords:** TIPS, Hemolytic anemia, Covered stent, Case report

## Abstract

**Background:**

In the 1990s, transjugular intrahepatic portosystemic shunts (TIPS) were performed using bare metal stents, and stent-induced hemolysis was a complication noted in 10% of patients. This was due to the mechanical stress created by turbulent flow from the uncovered interstices. Polytetrafluoroethylene (PTFE) stents came into regular use in the early 2000s becoming the standard equipment for TIPS placements, which are predominately covered. Due to this, stent-induced hemolysis has become a rare phenomenon.

**Case presentation:**

We describe a case of TIPS-induced hemolysis in a 53-years-old Caucasian female patient without cirrhosis. The patient had a history of heterozygous factor 5 Leiden mutation and abnormal lupus anticoagulant profile with development of a portal vein thrombus. She had undergone previous TIPS placement complicated by a TIPS thrombosis 3 years after initial placement requiring venoplasty and extension of the stent. Within one month, the patient developed hemolytic anemia with extensive evaluation that did not yield an alternative cause. Due to temporal association and clinical symptoms, the hemolytic anemia was attributed to the recent TIPS revision.

**Conclusion:**

This particular case of TIPS-induced hemolysis in a patient who does not have cirrhosis has not been previously described in the literature. Our case highlights that TIPS-induced hemolysis should be considered in anyone who could have potential underlying red blood cell dysfunction, not just those with cirrhosis. Further, the case demonstrates an important point that mild hemolysis (i.e., not requiring blood transfusion) can likely be managed conservatively, without stent removal.

## Introduction

Transjugular intrahepatic portosystemic shunt (TIPS) is a procedure which creates a shunt between a hepatic vein and a portal vein for portal decompression [1]. The main indications are to treat complications related to portal hypertension including but not limited to refractory ascites and variceal bleeding. Additionally, a TIPS can be used to re-establish blood flow in the setting of acute portal vein thrombus ultimately preventing such long-term consequences as cavernous transformation. When first gaining widespread use in the 1990s, TIPS were performed using bare metal stents. Stent-induced hemolysis was a complication noted in 10% of patients, thought due to the mechanical stress created by turbulent flow related to the uncovered interstices [2]. Polytetrafluoroethylene-covered stents came into regular use in the early 2000s and are the current standard of care for TIPS creation due to their improved patency [3]. Polytetrafluoroethylene (PTFE)-covered TIPS stents are predominately covered with a 2 cm uncovered portion designed to maintain patency of the contralateral portal vein. By reducing the overall bare metal surface area utilizing PTFE stents, stent-induced hemolysis has become a rare phenomenon.

We herein describe a case of new onset stent-induced hemolysis in a patient who underwent TIPS revision with a new PTFE stent placement.

## Case presentation

**Fig. 1:**
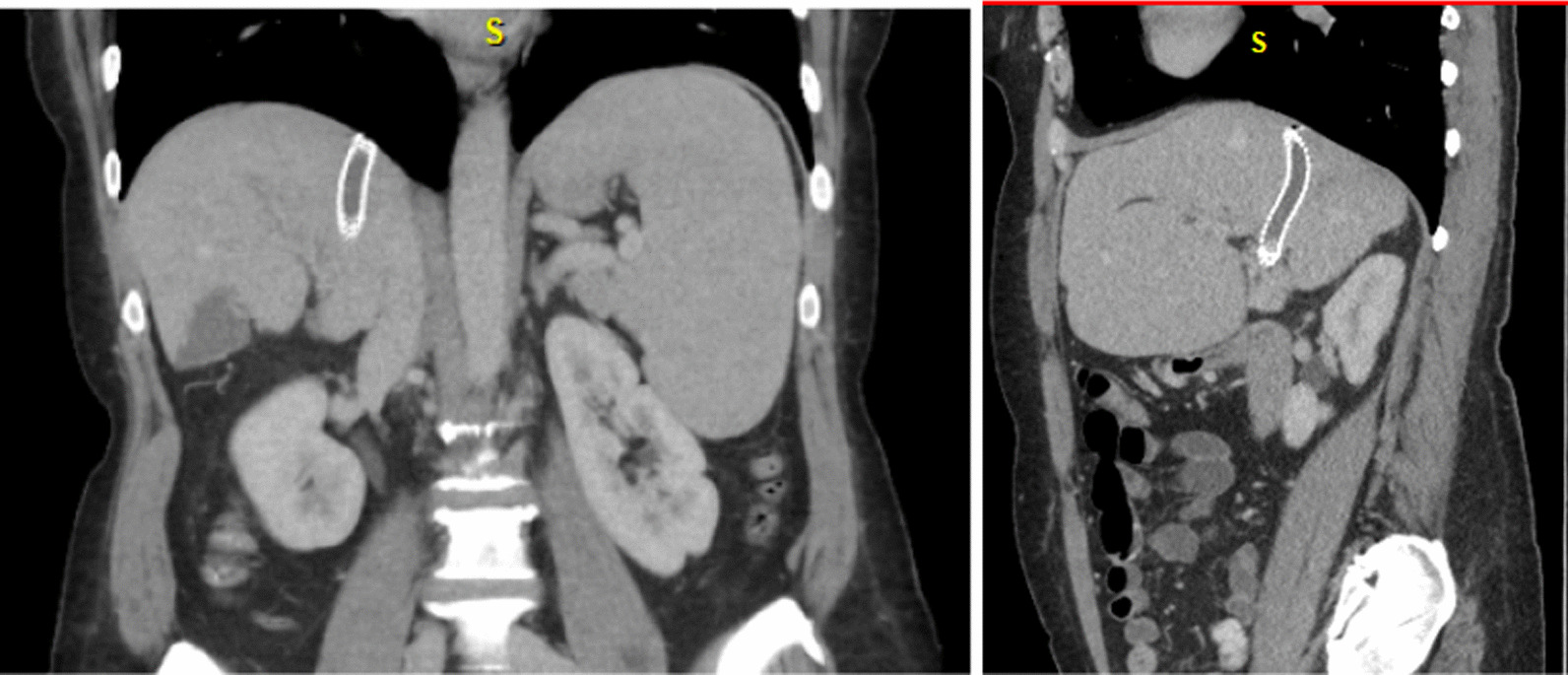
Coronal and sagittal view of computerized tomography abdomen/pelvis in the portal venous phase demonstrating thrombus within the stent

**Fig. 2:**
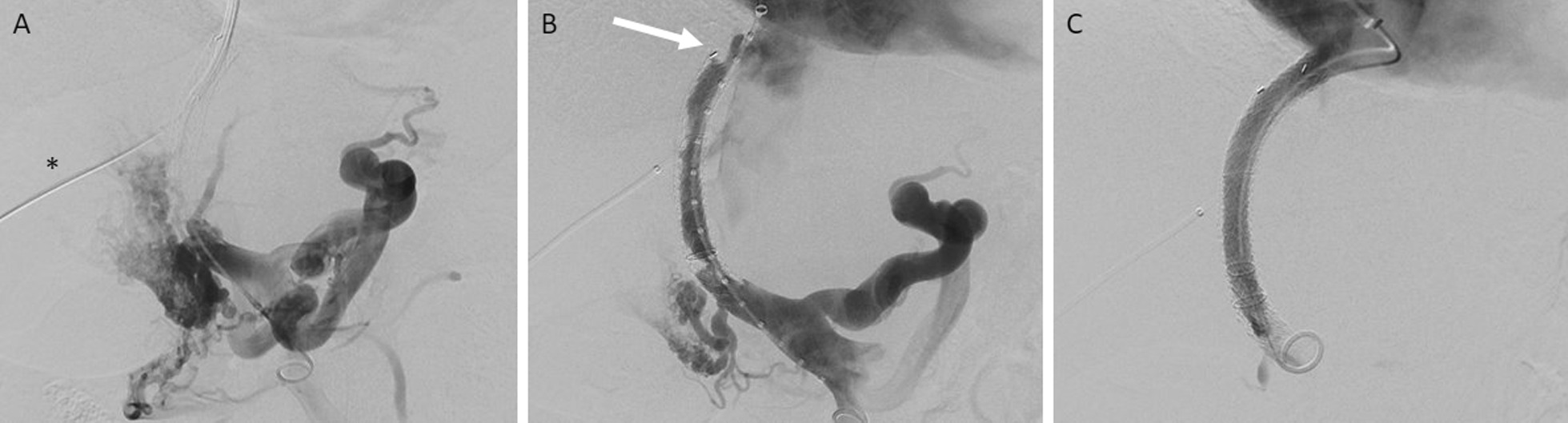
Transjugular intrahepatic portosystemic shunts revision with placement of additional stent graft towards the Inferior Vena Cava (IVC) confluence. **A** Initial transjugular intrahepatic portosystemic shunts-gram demonstrating complete occlusion of the transjugular intrahepatic portosystemic shunts with large varices. Note the transhepatic catheter (black asterisk) to the transjugular intrahepatic portosystemic shunts used to gain access to the occluded transjugular intrahepatic portosystemic shunts. **B** following venoplasty of the transjugular intrahepatic portosystemic shunts, there is reconstitution of low, however a persistent narrowing is present at the hepatic venous end of the transjugular intrahepatic portosystemic shunts (white arrow) with additional narrowing near the portal venous end. **C** Following placement of the additional stent graft, there is resolution of both narrowings with a resultant portosystemic gradient of 2

A 53-year-old Caucasian female with history of hemochromatosis and myeloproliferative (MF) neoplasm with myelofibrosis, MF-2. She was also diagnosed with heterozygous factor 5 Leiden mutation and abnormal lupus anticoagulant profile leading to portal vein thrombus. She had previously undergone TIPS placement for portal vein thrombosis with resultant abdominal pain which initially improved her symptoms. She had originally undergone TIPS in 2019 with placement of a PTFE-covered stent graft. One year later, she underwent revision whereby venoplasty was performed of the TIPS. This patient sought our care in 2022 due to worsening abdominal pain. The evaluation included a computerized tomography (CT) scan of the abdomen with contrast (Fig. 1) and a liver doppler ultrasound. Both studies demonstrated occlusion of the TIPS. She was referred to interventional radiology (IR) for revision. Given the TIPS thrombosis, a transhepatic puncture of the occluded portion of the TIPS was performed and a wire was advanced towards the right atrium. This wire was snared from a right internal jugular access such that access to the thrombosed TIPS was gained. Venogram confirmed occlusion of the TIPS (Fig. 2). This was initially treated with venoplasty. Subsequent venogram demonstrated a persistent narrowing at the hepatic venous end of the TIPS, which was treated by placing a longer stent within the existing stent, covering the stenosis and extending the TIPS to the Inferior Vena Cava (IVC) confluence. A 10 cm stent graft (8 cm covered, 2 cm uncovered) (Viatorr, Gore, Flagstaff, AZ) was utilized and dilated to 10 mm. The portosystemic gradient was reduced from 12 to 2 mmHg following the revision.

Three weeks later, the patient presented with dark urine and dyspnea on exertion and was found to have new onset hemolytic anemia (Table 1). She underwent an extensive evaluation for hemolytic anemia and ultimately the temporal association of recent TIPS revision and development of her symptoms led to the diagnosis. Follow-up liver ultrasound with doppler demonstrated mildly elevated velocities with patent TIPS shunt. The images were reviewed with IR who did not see any evidence of clinically significant TIPS dysfunction. The patient was managed conservatively with improvement in her lab work within 16 weeks post-TIPS revision and no development of subsequent symptoms (Table 1). Repeat liver ultrasound with doppler at that time demonstrated normal flow through the TIPS with resolution of the elevated velocities.

**Table 1:** Laboratory values: pre-transjugular intrahepatic portosystemic shunts, 3 weeks post-transjugular intrahepatic portosystemic shunts revision and 4 months post-transjugular intrahepatic portosystemic shunts revision

Labtest	Pre-TIPS	3 weeks post-TIPS revision	4 months post-TIPS revision
Hemoglobin (g/dL)	12.7	10.8 (11.6–15)	11.3
Reticulocytes (%)	–	3.63% (0.6–2.71)	–
Absolute reticulocytes × 10(9)/L	–	127.8 (30.4–110.9)	–
Total bilirubin (mg/dL)	0.7	3.1 (< 1.2)	2.3
Direct bilirubin (mg/dL)	–	0.5 (0.0–0.3)	0.6
Lactate dehydrogenase (U/L)	–	533 (122–222)	496
Haptoglobin (mg/dL)	–	< 14 (30–200)	21
Direct Coombs test	–	Negative	–

Labwork demonstrating evidence of hemolytic anemia with normal ranges listed. Comparison hemoglobin and total bilirubin pre-TIPS revision listed

## Discussion

Our case highlights a time-course that fits with TIPS-induced hemolysis, considered a rare phenomenon with covered stents. To our knowledge there are only a handful of case reports demonstrating post-TIPS hemolysis with Viatorr stents [4–6]. Our case is unique in that this patient was tolerating a TIPS covered stent graft without evidence of hemolytic anemia but following revision, developed evidence of hemolysis. In this case, with the revision, the covered stent graft was extended about 2 cm towards the IVC confluence. Viatorr stents, while predominantly covered, do have an uncovered bare metal end measuring 2 cm. There exists a ‘naked stent’ concept that uncovered stents can cause turbulent flow thereby inducing sheer stress on the red blood cells ultimately leading to hemolysis [2, 7]. The ‘naked stent’ concept has been derived from the phenomenon of intravascular hemolysis seen from turbulent flow due to mechanical aortic valves [2, 7]. This small, uncovered section of the Viatorr stent may predispose to turbulent flow. However, it is not clear if this contributed to hemolysis in our case as the uncovered portion of the stent was still deployed within the original TIPS stent graft.

One key difference as pointed out by Sanyal *et al*. is flow within the aorta is significantly higher compared to mesenteric venous flow and TIPS. They postulate that many patients who require TIPS have cirrhosis and as a result have inherent red blood cell dysfunction [2]. When combining the low-functioning red blood cells with sheer stress, there is an increased risk for hemolysis. At the time of TIPS revision for our patient, a transjugular liver biopsy was performed, showing no evidence of cirrhosis. Most likely, the underlying portal hypertension was due to a pre-hepatic etiology (i.e. chronic portal vein thrombosis). This points out another unique finding in our case—to our knowledge, this is the only published case of post-TIPS hemolysis in a non-cirrhotic patient suggesting there may be an additional unclear mechanism in place that contributed to the hemolytic anemia. The possibility of impaired red cell stability in the setting of myelofibrosis may have also contributed to the hemolytic anemia. Our patient was managed conservatively and now has compensated hemolytic anemia without any symptoms 4 months post-TIPS revision. This highlights an important point that mild hemolysis (i.e., not requiring blood transfusion) can likely be managed conservatively, without stent removal.

## Conclusions

This particular case of TIPS-induced hemolysis in a patient who does not have cirrhosis has not been previously described in the literature. Our case highlights that TIPS-induced hemolysis should be considered in anyone who could have potential underlying red blood cell dysfunction, not just those with cirrhosis. Further, the case demonstrates an important point that mild hemolysis can likely be managed conservatively, without stent removal.

## Data Availability

Not applicable.

## Abbreviations

TIPS: Transjugular intrahepatic portosystemic shunts
PTFE: Polytetrafluoroethylene
IR: Interventional radiology
